# Outcome of Endovascular and Open Treated Penetrating Aortic Ulcers

**DOI:** 10.1177/15266028241241205

**Published:** 2024-03-27

**Authors:** Marvin Kapalla, Joselyn Kröger, Rahul Choubey, Albert Busch, Ralf-Thorsten Hoffmann, Christian Reeps, Steffen Wolk

**Affiliations:** 1 Division of Vascular and Endovascular Surgery, Department of Visceral, Thoracic and Vascular Surgery, University Hospital Carl Gustav Carus, TU Dresden, Dresden, Germany; 2 Institute for Diagnostic and Interventional Radiology, University Hospital Carl Gustav Carus, TU Dresden, Germany

**Keywords:** penetrating aortic ulcers, endovascular treatment penetrating aortic ulcers, surgical treatment penetrating aortic ulcers, mortality, paraplegia, intramural hematoma

## Abstract

**Purpose:**

Penetrating aortic ulcer (PAU) is a rare etiology of acute aortic syndrome. Few studies exist regarding the perioperative outcome. The aim was to analyze clinical outcome and risk factors of mortality in this treatment population.

**Methods:**

Retrospective, monocentric study from 2010 to 2021. Clinical data of endovascular or open treated PAU were analyzed. In-hospital mortality was selected as the primary study endpoint. Angio-morphologies were analyzed and risk factors for mortality were identified by using univariate analysis.

**Results:**

Overall, 133 patients were identified. 29% (n=38) of patients presented symptomatically. In 64% (n=85), the PAU was localized in the thoracic aorta. On average, PAUs had a depth of 15.4±10.1 mm and a width of 17.9±9.6 mm. The patients had a median of 2 (95% confidence interval [CI]=2-3) high-risk features (HRF) as PAU depth >10 mm, PAU width >20 mm, aortic diameter >40 mm, symptomatic, intramural hematoma (IMH), pleural effusion. Significantly more HRF were observed in symptomatic patients (p=0.01). 53% (n=71) of patients were treated with thoracic endovascular aortic repair (TEVAR), 41% (n=54) by endovascular aortic repair (EVAR), and 6% (n=8) by open surgery. A hybrid procedure with cervical debranching was performed in 16% (n=21) and complex endovascular repair with fenestrated or branched endografts in 15% (n=20). Overall, complications greater than grade II according to the Clavien-Dindo classification occurred in 19% (n=25) and of the patients. In-hospital mortality manifested in 6% (n=8). Factors associated with increased mortality were the diameter of the aorta >40 mm (88% vs 39%, p=0.03), as well as symptomatic patients (63% vs 26%, p=0.04), coincident IMHs (38% vs 10%, p=0.05), and complex endovascular procedures (50% vs 50% p<0.01). Penetrating aortic ulcer width >20 mm tended to show higher mortality (75% vs 40%, p=0.06). Routine follow-up was available for 89% (n=117) for a median of 39 months (95% CI=25-42). One-year and 5-year survival were 83% and 60%, respectively, with 1 aortic pathology-related death.

**Conclusions:**

Treatment of PAU is associated with an acceptable perioperative morbidity and mortality. Risk factors associated with increased mortality are an elevated aortic diameter, the presence of IMHs, clinical symptomatology at presentation, and complex endovascular procedures.

## Introduction

As described by Stanson et al^1^ in 1986, the penetrating aortic ulcer (PAU) is defined as an ulcerative defect of the aortic wall originating from the intima and penetrating the media. The clinical course is variable, PAUs can remain stable, enlarge, or progress to intramural hematoma (IMH), dissection, pseudoaneurysm, or even rupture.^2,3^ Often diagnosed asymptomatically as incidental findings on computed tomography angiography (CTA), PAUs belong to the group of acute aortic syndromes when symptomatic manifestation occurs.^4^ Studies reported that PAUs were causative for 2% to 7% of acute aortic syndromes (AAS) with rupture rates up to 38%.^5,6^

As also observed, the potential for progression of the lesion is higher than in other aortic diseases such as aneurysms or dissections. Furthermore, this progression cannot be influenced effectively by antihypertensive or β-blocking therapy.^7^

Nevertheless, the indications for the invasive treatment of PAUs, especially in asymptomatic patients, are not well-defined, controversial, and limited to late outcome data.^8,9^ Although PAUs as localized pathologies, they are often manageable by simple endovascular procedures. However, PAUs in more delicate localizations, such as the aortic arch or the thoracoabdominal transition zone, can require much more complex treatment. To date, there are only a few publications about the outcomes of endovascular and/or open surgical treatment with correspondingly low evidence.^3,10,11^ Even so, the possible treatment outcomes of these pathologies should be included in the decision-making process, especially if the primary indication is controversial and indeterminate. The study aimed to evaluate the results of multifaceted surgically-treated patients during the last 10 years and to analyze clinical outcomes and risk factors for mortality.

## Methods

### Data Collection and Study Population

All patients consecutively treated for PAUs either by an open surgical or an endovascular approach between January 2010 and January 2021 in the Department of Vascular and Endovascular Surgery at the Carl Gustav Carus University Hospital, Dresden were analyzed. The data for each case were obtained retrospectively based on electronic patient records. Inclusion criteria were diagnosis of PAU by CTA followed by consecutive surgical therapy. In CTA PAU disease was defined as the focal outpouching of contrast through an area of intimal calcification and severe atherosclerotic disease.^2,12^ Exclusion criteria were conservatively treated PAUs, PAUs of the ascending aorta as well as mycotic lesions and ulcer-like projection (ULP). Radiographic exclusion criteria were ulcerated plaque without proof of intimal disruption.^13^ Further data like demographic data, comorbidities, clinical presentation, radiologic data (width, depth, length, total aortic diameter at the level of the PAU pathology, eccentricity index), treatment modalities, complications, length of hospital stay, and follow-up examinations were collected.

### Ethics Approval

All procedures in studies involving human participants complied with the ethical standards of the institutional research committee.

Under the guidelines for research on human subjects, the local ethics committee at the Technische Universität Dresden approved the study (decision number EK 297072018). The ethics committee is registered as an institutional review board (IRB) at the Office for Human Research Protections (OHRP) (registration numbers IRB00001473 and IORG0001076).

### Indications and Surgical/Interventional Technique

**Figure 1.**
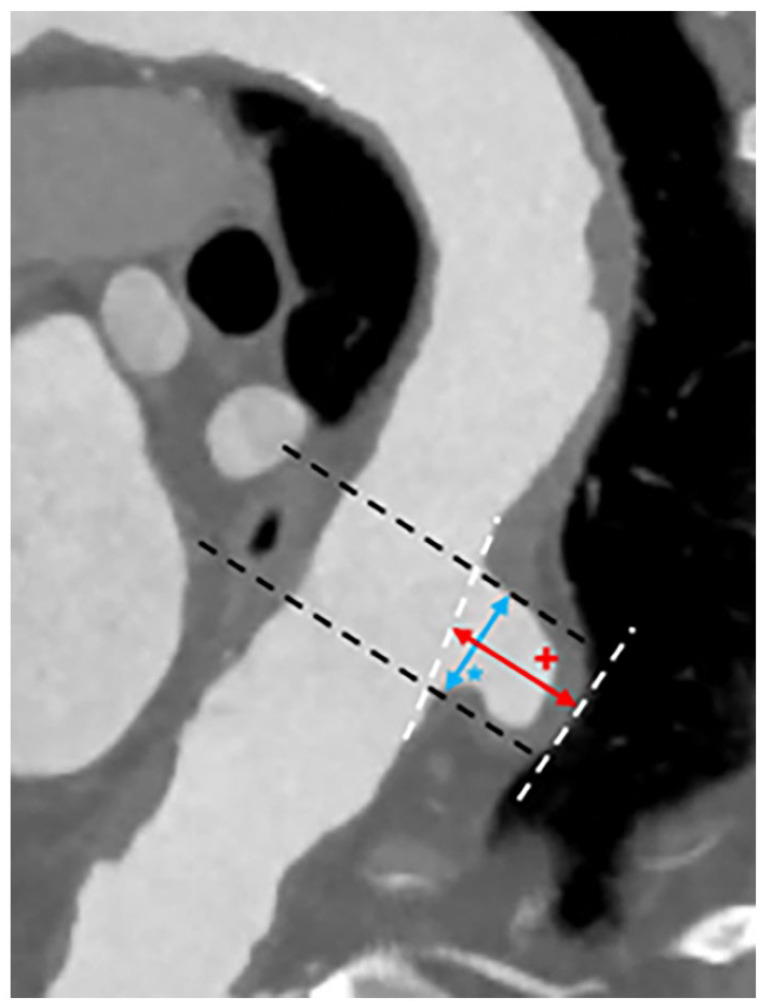
PAU measurement (CTA): PAU depth (red +), PAU wide (blue*).

Indications for immediate interventional/surgical treatment were set in the presence of clinical symptoms or hemodynamic instability. For asymptomatic patients, an interdisciplinary vascular board (radiologists, angiologists, vascular surgeons) decided whether patients underwent medical or interventional/surgical treatment depending on the PAU’s morphology (Figure 1), clinical status and surgical risk factors of the patients. Indications for invasive treatment were given by highly eccentric PAU lesions with at least depth >10 mm, width >20 mm, and rapid increase in the size of the PAU (short or long term) or morphological changes (new IMH, enlarging pseudoaneurysm). In case of coincident IMHs, the indication for treatment of IMH was set in patients with early expansion of the pathology, persistent symptoms despite optimal conservative therapy, IMH thickness >10 mm, and secondary dilatations or dissections. All patients with an IMH are monitored closely by CT.

General anesthesia was used in all patients. During open and endovascular approach, systemic heparin was administered to maintain the activated clotting time between 250 and 300 seconds. The activated clotting time was checked in 30-minute intervals. Lumbar drains were placed in elective cases for the prevention of spinal cord ischemia at the discretion of the surgeon, with general indications for patients requiring long segment repair, repair in the distal thoracic aorta especially in patients who had prior history of abdominal aneurysm repair.

In open surgical management, patients received aortic replacement using a Dacron prosthesis. In cases involving thoracic or thoracoabdominal replacement, an extracorporeal perfusion support was performed by extracorporeal membrane oxygenator for distal and selective reno-visceral perfusion.

In endovascular approaches, percutaneous access was obtained through both femoral arteries. In complex endovascular procedures, access via the axillary artery was also established if deemed necessary. Device positioning and deployment were guided by angiographic landmarks. Devices utilized included i.a. TAG (WL Gore, Flagstaff, Arizona), Zenith Alpha (Cook, Bloomington, Indiana), Endurant (Medtronic, Minneapolis, Minnesota), and custom-made devices. Stent-graft sizing was performed using CTA scan measurements with 3-dimensional reconstruction. The stent grafts selected were oversized by 10% of the diameter of the aorta. We routinely performed revascularization of the left subclavian artery using a left-carotid-to-subclavian artery bypass if overstenting was required to achieve an adequate proximal landing zone. Molding with a compliant balloon of attachment and overlap zones were performed routinely, except for IMH. In endovascular repair, technical success was defined as scheduled placement of an aortic stent graft and exclusion of the target aortic pathology without evidence of type I or III endoleaks in accordance with reporting standards for endovascular aortic aneurysm repair.^14^ All endovascularly treated patients received routine CTA on the first postoperative day to confirm proper stent-graft implantation.

In our center’s general treatment decision-making, an “endovascular first” approach is adopted. However, in cases involving complex anatomies, especially in emergency situations, an open procedure is recommended when clinically suitable.

When a reno-visceral stent or chimney was implanted, patients subsequently received acetylsalicylic acid (ASA) 100 mg and clopidogrel 75 mg for 3 months. Thereafter, aspirin only was administered for life-long use. Otherwise, further anticoagulation and antiplatelet therapy was based on the patients’ respective cardiac and vascular comorbidities.

Routine follow-up consisted of clinical examination, duplex sonography and/or CTA every 3 and 6 months during the first year and at least annually after that.

### Outcome Parameters and Definitions

For the surgical-anatomical classification of the aorta into its 5 segments, we use the classification according to Heberer.^15^ The thoracic aorta begins with the ascending aorta at the supracoronary junction extending to the innominate artery (segment I), followed by segment II to the left common carotid artery (LCCA). Segment III extends distal to the LCCA including the left subclavian artery and to the thoracoabdominal transition above the coeliac trunk.

The abdominal aorta includes the distal portion of segment III extending to just below the diaphragm, the visceral segment IV, and the infrarenal segment V.^15^ The primary endpoint of this study was the in-hospital mortality. Secondary outcome parameters were primary technical success, morbidity, and overall survival. In accordance with reporting standards for endovascular aortic aneurysm repair, technical success was defined as correct placement of the main body and exclusion of the target pathology without evidence of type I or III endoleaks. Assisted primary success was defined if further unplanned treatment procedures (eg, due to a type Ia endoleak) were necessary during the primary procedure for elimination of the target pathology.^14^ Complications and postoperative course were categorized according to the Society for Vascular Surgery (SVS) reporting standards for complex endovascular aortic repair and the Clavien-Dindo classification and recorded when they exceeded grade I.^14,16^ The early postprocedural period was defined as first 30 days postoperatively, or if patients remained in hospital for a longer duration, it extended until the period of discharge. The follow-up period was defined as the period from hospital discharge to the date of the last available clinical examination. Missing follow-up information was completed via telephone interviews with patients or primary care physicians. Risk factors for in-hospital mortality were identified using univariate analysis.

### Statistical Analysis

Statistical analysis was performed using IBM SPSS for Windows, Version 21.0 (IBM Corp, Armonk, New York). All clinical characteristics were grouped to build categorical or nominal variables. Dichotomous variables were recorded as absolute frequencies (number of cases) and relative frequencies (percentages). Continuous data are presented as mean and standard deviation; non-symmetrical with median and interquartile range (IQR). Pearson’s chi-squared or Fisher’s exact test was used to analyze categorical variables. Differences between means were tested with *t*-test or Mann-Whitney *U*-test. Survival data were analyzed using Kaplan-Meier estimates and differences were appointed by the log-rank test. A 2-sided p<0.05 was considered statistically significant.

## Results

### Study Population and Patient Characteristics

The patient cohort comprised 133 patients (male n=105, 79%) with a mean age of 72.7±8.4 years. Penetrating aortic ulcer diagnosis was made incidentally in 32% (n=42) and in 68% (n=89) after suspicion. Additional coincident aortic pathologies in the initial CTA were dissections in 14% (n=19), aneurysm in 55% (n=73) and IMHs in 11% (n=14) of the patients. Common comorbidities and risk factors were hypertension (82%), nicotine abuse (29%), chronic kidney injury (glomerular filtration rate (GFR) <30 mL/min/1.73 m^2^) (33%), diabetes mellitus (25%), and congestive heart failure (New York Heart Association NYHA II) (15%) (Table 1).

**Table 1.** Demographic and Clinical Data.

Variable^a^	n=133 (%)
Demographic data	
Age (years)	72.7 ± 8.35
Sex (male/female)	105/28 (79/21)
Risk factors and comorbidities	
Chronic kidney disease^b^	45 (34)
Heart failure (>NYHA II)	20 (15)
Atrial fibrillation	30 (23)
Hypertension	109 (82)
CHD	26 (20)
Myocardial infarction	13 (9)
Diabetes mellitus	33 (25)
COPD	13 (10)
Nicotine abuse	39 (29)
Coincident aortic pathologies	
Dissection	19 (14)
IMH	15 (11)
Aneurysm (<50 mm)	73 (55)

Abbreviations: CHD, coronary heart disease; COPD, chronic obstructive pulmonary disease; IMH, intramural hematoma NYHA, New York Heart Association.

a Continuous data presented as mean±standard deviation.

b GFR <30 mL/min/1.73 m^2^.

### Clinical Presentation and Morphology

Overall, 28.6% (n=38) of the patients presented with a thoracic or abdominal symptomatology. Contained rupture was diagnosed in 13.6% (n=18) of the patients, and all except 1 patient (0.8%) was hemodynamically stable. The etiology of PAUs was arteriosclerotic in all cases.

In 63.9% (n=85) of the cases, the PAU was located in the thoracic and in 36.1% (n=48) in the abdominal aorta. Symptomatic showed higher frequency of localization in the thoracic aorta (81.6%, n=31 vs 18.4, n=7; p=0.009). Further comparison of the clinical data between symptomatic and asymptomatic patients showed no significant differences (Table 2).

**Table 2.** Comparison of Clinical Data From Symptomatic Patients.

Variable^a^	Symptomatic	Asymptomatic	p
	n=38 (%)	n=95 (%)	
Age (years)	73 ± 8	74 ± 9	0.45
Sex (male/female)	28/10 (74/26)	77/18 (81/19)	0.2
Chronic kidney disease^b^	9 (26)	36 (38)	0.24
Heart failure (>NYHA II)	4 (11)	16 (17)	0.49
Atrial fibrillation	7 (18)	23 (24)	0.67
CHD	5 (13)	21 (22)	0.36
Diabetes mellitus	6 (16)	27 (28)	0.22
COPD	3 (8)	10 (11)	0.78
Morphologies			
Depth (mm)	15.8 ± 10.7	14.2 ± 8.3	0.66
Length (mm)	28.8 ± 16.7	26.9 ± 13.5	0.54
Width (mm)	19.8 ± 12.1	17.4 ± 8.5	0.71
Eccentricity index^c^	1.8 ± 1	1.78 ± 0.8	0.77
Aortic diameter (mm)	41.3 ± 13.1	41.1 ± 13.6	0.96
Localization			
Thoracic aorta	31 (82)	54 (57)	<0.01
Abdominal aorta	7 (18)	41 (43)	
High-risk features			
PAU depth >10 mm	24 (63)	63 (66)	0.65
PAU width >20 mm	16 (42)	40 (42)	0.61
Aortic diameter >40 mm	13 (34)	42 (44)	0.49
Pleural effusions	4 (11)	0	0.01
IMH	9 (24)	6 (7)	0.02
∑	3 [95% CI*** 3-4]	2 [95% CI*** 2-3]	<0.01

Abbreviations: CHD, coronary heart disease; COPD, chronic obstructive pulmonary disease; CI, confidence interval; IMH, intramural hematoma; NYHA, New York Heart Association; PAU, penetrating aortic ulcer.

a Continuous data presented as mean±standard deviation.

b GFR <30 mL/min/1.73 m^2^.

c CI, confidence interval.

According to the Heberer classification, 6% (n=8) of the lesions were located in segment II, 58% (n=77) in segment III, 10% (n=13) in IV, and 26% (n=35) in V. Multiple PAUs were seen in 15.8% (n=21) of the patients (Table 3). On average, the PAUs had a depth of 15.4 mm±10.1 mm, width of 17.9 mm±9.6 mm, and a length of 27.4 mm±14.4 mm. The aortic diameter at the level of the lesion in the cohort was 41.2 mm±13.5 mm. Overall, 73% (n=40) of patients had diameter of the thoracic aorta above 40 mm at the level of the PAU and 17% (n=15) of patients in the abdominal aorta. The determined eccentricity index (PAU diameter/length) was on average 1.78±0.85. The coincident IMHs exhibited a mean length of 178.5 mm±65.7 mm and a thickness of 7.2 mm±4.1 mm.

**Table 3.** PAU’s Localization and Morphologies.

Variables^a^	n=133 (%)
Localization (according to Heberer^b^)	
Segment II	8 (6.1)
Segment III	77 (57.9)
Segment IV	13 (9.8)
Segment V	35 (26.4)
Thoracic aorta	85 (63.9)
Abdominal aorta	48 (36.1)
Morphologies	
Depth (mm)	15.4 ± 10.1
Length (mm)	27.4 ± 14.4
Width (mm)	17.9 ± 9.6
Eccentricity index^c^	1.78 ± 0.85
Aortic diameter (mm)	41.2 ± 13.5
Multiple PAUs	21 (15.8)
n=2	18 (13.5)
n=3	1 (0.8)
n=4	2 (1.5)

a Continuous data presented as mean±standard deviation

b Pandey and Sharma.^8^

c Aortic diameter/length.

Thrombus filling of the PAUs was present in 27.8% (n=37). Patients had a median of 2 (95% confidence interval [CI]=2-3) high-risk features (HRFs) (PAU depth >10 mm, PAU width >20 mm, aortic diameter >40 mm, symptomatic, IMH, pleural effusion)^17^ (Table 4). Symptomatic patients showed significantly more HRFs than asymptomatic patients (3 [95% CI=3-4] vs 2 [95% CI=2-3]; p=0.01).

**Table 4.** High-Risk Features.

Variables	n=133 (%)
Feature	
Symptomatic	38 (28.6)
Pleural effusion	4 (3)
IMH	15 (11.3)
Depth >10 mm	87 (65.4)
width >20 mm	56 (42.1)
Aortic diameter >40 mm	55 (42)
Quantity	
n=0	24 (18)
n=1	26 (19.5)
n=2	37 (27.8)
n=3	32 (24.1)
n=4	13 (9.8)
n=5	1 (0.8)

Abbreviation: IMH, intramural hematoma.

### Treatment

The median time from initial diagnosis to surgical treatment was 10 days (95% CI=6-24 days). In 36.8% (n=49), at least 1 additional CT imaging was performed before treatment. The indication for treatment due to a progression of the PAU was given in 75% (n=40) of these patients. Early/elective treatment during the primary hospital stay was performed in 80% (n=106) of the cases.

53% (n=71) of patients were treated by thoracic endovascular aortic repair (TEVAR), 41% (n=54) by endovascular aortic repair (EVAR), and 6% (n=8) by open surgery. Whereas a hybrid procedure was used in 15.8% (n=21) (Table 5). The indications for open surgery were emergency treatments in 5 patients (n=3 contained rupture and n=2 symptomatic) with problematic anatomy for rapid endovascular treatment, unsuitable access ways for endovascular treatment (narrow vessel diameters and severe calcification of the pelvic axis) in 2 patients and in 1 patient due to dilatation of the aorta in the reno-visceral segment and location of the PAU in this area. Cervical debranching (carotid subclavian bypass) was performed in 15% (n=20) of the procedures and complete reno-visceral debranching in 1 (1%) as part of an aortic hybrid procedure. Complex endovascular repair with fenestrated or branched endografts was performed in 15% (n=20) patients. This included n=4 (3%) aortic arch endografts (3 double-branched and 1 triple-branched), as well as 9 (7%) quadruple branched or fenestrated aortic stent grafts. The primary-assisted technical success was 100%. Postoperative endoleak rate was 6.4% (n=8/125) confirmed by routinely performed postoperative CTA and 75% (n=6) of all endoleaks occurred in the cases of complex endovascular repair. Due to complex endovascular repair, type III endoleak was most common in 6 (4.8%) patients (n=3 IIIa, n=2 IIIc, n=1 IIIb) followed by Ia (0.8%, n=1) and Ib (0.8%, n=1) (Table 5). All type I and III endoleaks were successfully treated and occluded endovascularly.

**Table 5.** Treatment Modalities.

Variables	n=133 (%)
Open surgery	8 (6)
Thoracic with ECMO	1 (1)
Abdominal	5 (4)
Thoracoabdominal with ECMO	2 (2)
Endovascular	125 (94)
TEVAR	71 (53)
Cervical debranching	20 (15)
f/b-TEVAR	8 (6)
Branched aortic arch endografts	4 (3)
EVAR	54 (41)
Visceral debranching	1 (1)
Chimney	3 (2)
f/b-EVAR	9 (7)

Abbreviations: ECMO, extracorporeal membrane oxygenation; f/b-EVAR, fenestrated-branched endovascular aortic repair.

### Patient Outcome

Overall, complications occurred in 18.8% (n=25) of patients. According to the Clavien-Dindo classification, 12.8% (n=17) patients developed complications grade IIIa to VIb (Table 6). Surgical revision was necessary for 15.8% (n=21) of the patients. Indications for this included wound healing disorders/hematomas (n=9; 42.8%), endoleaks (n=8; 38.1%), femoral artery pseudoaneurysm (n=3; 14.3%), and an aortic graft infection (n=1; 4.8%). No permanent paraplegia occurred, but 1 patient suffered from temporary paraplegia after TEVAR and fully recovered after lumbar drainage and permissive hypertension. Perioperative minor stroke occurred in 2 patients (1.4%, 2-fold branched aortic arch endografts) due to significant atheromatosis of the aortic arch. One patient suffered from an acute intraoperative arterial embolism of the lower extremity, with the necessity of open embolectomy. Acute renal failure necessitating dialysis developed in 2 (1.4%) with an additional 2 patients experiencing septic multiorgan failure (1.4%).

**Table 6.** Endoleaks and Complications.

Variables	n (%)
Endoleak postoperative^a^	8 (6.4)
Ia	1 (1)
Ib	1 (1)
III	6 (4.8)
Complications^b^	25 (18.8)
IIIa	2 (1.5)
IIIb	8 (6)
Iva	5 (3.7)
IVb	2 (1.5)
In-hospital mortality	8 (6)

a n=125 endovascular treated patients.

b According to the Clavien-Dindo classification^9^ in n=133 patients.

The 30-day and in-hospital mortality manifested at 6% (n=8). The mean length of in-hospital stay was 15±13 days overall and 4±8 days for the intensive care unit (ICU).

**Figure 2.**
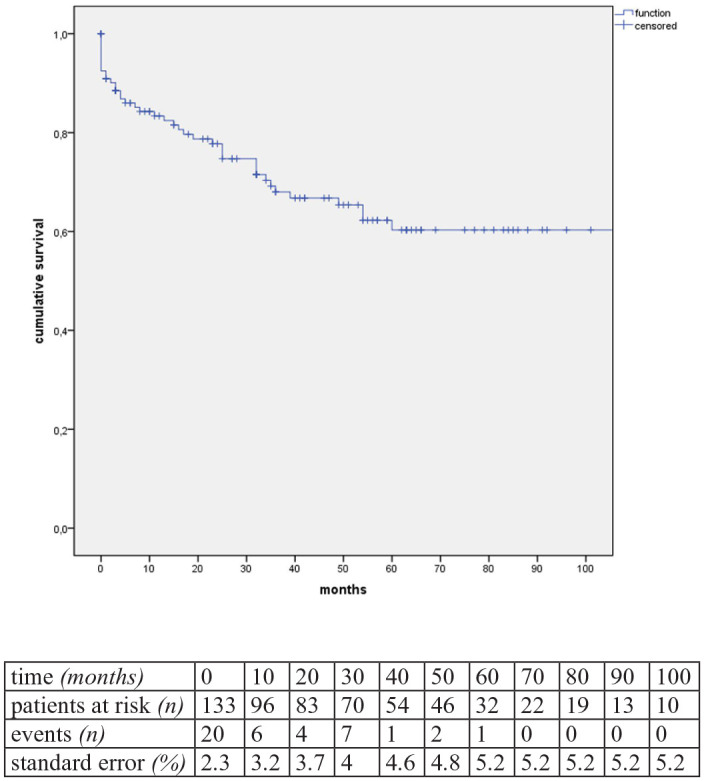
Kaplan-Meier estimates of overall survival (estimator for median survival: 94.5 months, standard error [SE]: 6.8%, 95% CI=81-105 months).

Systematic follow-up was available for 89% (n=117) for a median of 39 months (95% CI=25-42). In total, there were 36 late deaths (28.8%), of which 1 was aorta-related due to stent-graft infection. Estimated Kaplan-Meier for 1-year and 5-year survival was 83% (standard error=3.2%) and 60% (standard error=5.2%) (Figure 2). During follow-up, no new PAUs or significant progression of the multiple PAUs (n=21) were observed.

### Risk Factors for Mortality

Symptomatic patients presented with significantly higher mortality (62.5% vs 26.4%; p=0.04) as well as patients with contained rupture (62.5% vs 10.4%; p=0.02). Patients with a PAU width >20 mm tended to have higher mortality (75% vs 37.5%; p=0.06). In contrast, PAUs with a depth >10 mm (87.5% vs 64%; p=0.16) as well as a location in the thoracic aorta (75% vs 63.2%; p=0.5) exhibited no effect on mortality. Similarly, the treatment modality (surgical vs hybrid vs open) showed no significant difference in survival (p=0.61). There were no significant differences in endovascular treatment (TEVAR vs EVAR; p=0.45), but patients with complex endovascular repair (including f/b-TEVAR, f/b-EVAR, and chimney EVAR) showed significantly worse survival (80% vs 20%; p<0.01).

Furthermore, univariate analysis identified an aortic diameter >40 mm (87.5% vs 39.2%; p=0.03), as well as coincident IMHs (37.5% vs 9.6%; p=0.05) with increased mortality. Also, deceased patients showed significantly more frequent pleural effusions (37.5% vs 1.6%; p=0.02), a history of heart failure (50% vs 12.8%; p=0.01), and atrial fibrillation (50% vs 20.8%; p=0.04) (Table 7).

**Table 7.** Factors Associated With In-Hospital Mortality in n=133 Patients.

Variable	Deceased	Survived	p
	n=8 (%)	n=125 (%)	
Heart failure (>NYHA II)	4 (20)	16 (80)	0.01
Atrial fibrillation	4 (13.3)	26 (86.7)	0.04
CHD	3 (37.5)	23 (18.4)	0.09
Contained rupture	5 (27.8)	13 (72.2)	0.02
Treatment			
Operative	1 (12.5)	7 (87.5)	0.61
Hybrid	1 (4.8)	20 (95.2)	
Endovascular	6 (5.8)	98 (94.2)	
TEVAR	4 (8.5)	65 (91.5)	0.45
EVAR	2 (5.1)	56 (94.9)	
Complex endovascular repair^a^	4 (20)	16 (80)	<0.01
Localization			
Thoracic aorta	6 (7.1)	79 (92.9)	0.5
Abdominal aorta	2 (4.2)	46 (95.8)	
High-risk features			
PAU depth >10 mm	7 (8)	80 (82)	0.16
PAU width >20 mm	6 (10.7)	50 (89.3)	0.06
Aortic diameter >40 mm	7 (12.5)	49 (87.5)	0.03
Pleural effusions	2 (50)	2 (50)	0.02
IMH	3 (20)	12 (80)	0.05
Symptomatic	5 (13.2)	33 (86.8)	0.04

Abbreviations: CHD, coronary heart disease; IMH, intramural hematoma; NYHA, New York Heart Association; PAU, penetrating aortic ulcer.

a Including f/b-TEVAR, f/b-EVAR, and chimney EVAR.

## Discussion

This study demonstrates that invasive treatment of PAU by endovascular and open approach is associated with an acceptable perioperative morbidity and mortality in a large cohort of 133 patients.

Evidence for the treatment of PAUs is limited.^10,11^ Considerable controversy exists regarding the natural history of these diseases and indications for treatment.^17^ Due to the lack of data, current guidelines do not provide specific treatment recommendations, especially for patients with asymptomatic PAU.^18^ The results of operative and endovascular treatment are limited by small series.

In our center, a total of 133 patients with PAU were treated, encompassing 29% (n=38) symptomatic cases of which 13.6% (n=18) showed contained rupture. Consistent with the recommendations of many authors, the indication for treatment was set in all symptomatic patients as well as in asymptomatic patients with HRFs.^2,8,17,19^ These HRFs like IMH, PAU depth >10 mm and wide >20 mm were previously demonstrated in studies forecasting disease progression.^8,12^ In particular, PAU rupture risk is directly related to ulcer depth, as reported in current guidelines from the SVS and Society of Thoracic Surgeons (STS).^20^ Moreover, in our study, HRFs were more often found in symptomatic patients, and these symptomatic patients showed a worse outcome (13.2% vs 3.2%; p=0.04). However, the potential predictors of PAU progression were not evaluated in this study because only surgically-treated patients were included.

As described by Patel et al^3^ and Bellomo et al,^21^ especially the combination of IMH and PAU was associated with increased mortality (20% vs 9.6%; p=0.05). So far, the causal relationship between PAU and IMH has not been established.^18^ A recent study also reported increased rates of stent-graft–induced new entries (SINEs) in patients treated due to PAU and IMH.^22^ Nevertheless, the combination of PAU and coincident IMH in our cohort (10.5%) was low in comparison to other studies, and no SINE was observed during follow-up examinations.

Overall, an early treatment was set in 80% of the cases. Only 20% (n=27) were treated during the follow-up due to a progression in the course imaging. In contrast to other authors, we did not find a significant difference in mortality between primary surgical and secondary surgical (=primary medical) treatment.^3,22,23^ In our institution, the “endovascular first approach” is the method of choice for anatomically suitable patients. Due to the meanwhile widespread availability and less invasive complex, endovascular procedures with fenestrated or branched endografts were performed in 15% (n=20) patients in morphologically elaborately localized PAUs. Owing to the complex and technically demanding procedures, these patients showed an increased mortality rate.^24,25^ Only 8 patients (6%) received open repair due to anatomical inaccessibility or emergencies. As expected, and already shown by other authors, mortality in patients undergoing open surgery was higher, but on a not significant level.^8,17,23^ Indeed, the direct comparison between open and endovascularly treated patients is not appropriate given the negative selection bias for surgically-treated patients due to their higher risk profile and unfavorable anatomy.^8^

Even though PAU is commonly observed as a segmentally localized wall lesion and thus an ideal target for stent grafting, we observed a postoperative endoleak rate of up to 6.4% (n=8). These can be attributed to the complex endovascular procedures for anatomically challenging pathologies in our cohort. This made revisions necessary in all 8 patients (6.4%) due to type I and type III endoleaks, which could be successfully occluded. We usually do not wait for a spontaneous occlusion of these endoleaks and subject the patients to short-term CTA follow-ups. Fortunately, both endoleaks and revisions had no effect on survival.

In-hospital mortality manifested at 6% (n=8) and is comparable to previously published series.^8^ As expected, patients with a contained rupture showed significantly worse survival. Also, additional HRFs such as hemorrhagic pleural effusions, coincident IMH’s, and an aortic diameter >40 mm were associated with significantly worse survival.

As known, consistent with our investigation, the majority of PAUs tend to affect the descending thoracic aorta.^8,22^ Furthermore, we observed that these patients presented significantly more frequent symptomatic (81.6%, p=0.009) and tend to have worse survival (p=0.04.). Therefore, asymptomatic patients with a thoracic aortic PAU outside an indication for operative treatment might benefit from more frequent surveillance.^8^

During follow-up examinations, we observed 36 late deaths (29%), of which 1 was related to a stent-graft infection. Estimated Kaplan-Meier 1-year and 5-year survival were remarkably low with 83% and 60%. As described in the latest review from Pandey and Sharma,^8^ late mortality is irrespective of the treatment modality, given the fact of the significant high cardiovascular comorbidities, which already were associated with higher in-hospital mortality.^17,26^

This study has several limitations. It is a retrospective single-center study, generating bias linked to retrospective data collection. Furthermore, during the long study period, there has been progress in medical therapy and endovascular technology that may have affected patients’ outcomes. Finally, we also included ruptured and morphological complex PAUs, leading to heterogeneity and deterioration of outcome results. Nevertheless, in our opinion, these entities also belong to be reported in the context of PAU treatment.

## Conclusions

Invasive treatment of PAU is associated with an acceptable perioperative morbidity and mortality. Although the PAU often occurs as a localized lesion, complex anatomies can be challenging and requiring more complex procedures, which are associated with increased mortality. Risk factors for increased in-hospital mortality are elevated aortic diameter, the presence of IMHs, clinical symptomatology at presentation, and complex endovascular procedures.
